# RHBDF2-Regulated Growth Factor Signaling in a Rare Human Disease, Tylosis With Esophageal Cancer: What Can We Learn From Murine Models?

**DOI:** 10.3389/fgene.2018.00233

**Published:** 2018-07-04

**Authors:** Vishnu Hosur, Michelle L. Farley, Benjamin E. Low, Lisa M. Burzenski, Leonard D. Shultz, Michael V. Wiles

**Affiliations:** The Jackson Laboratory, Bar Harbor, ME, United States

**Keywords:** Rhbdf2, TOC, EGFR, ADAM17/TACE, AREG, CRISPR-Cas9

## Abstract

Tylosis with esophageal cancer syndrome (TOC) is a rare autosomal dominant proliferative skin disease caused by missense mutations in the rhomboid 5 homolog 2 (RHBDF2) gene. TOC is characterized by thickening of the skin in the palms and feet and is strongly linked with the development of esophageal squamous cell carcinoma. Murine models of human diseases have been valuable tools for investigating the underlying genetic and molecular mechanisms of a broad range of diseases. Although current mouse models do not fully recapitulate all aspects of human TOC, and the molecular mechanisms underlying TOC are still emerging, the available mouse models exhibit several key aspects of the disease, including a proliferative skin phenotype, a rapid wound healing phenotype, susceptibility to epithelial cancer, and aberrant epidermal growth factor receptor (EGFR) signaling. Furthermore, we and other investigators have used these models to generate new insights into the causes and progression of TOC, including findings suggesting a tissue-specific role of the RHBDF2-EGFR pathway, rather than a role of the immune system, in mediating TOC; and indicating that amphiregulin, an EGFR ligand, is a functional driver of the disease. This review highlights the mouse models of TOC available to researchers for use in investigating the disease mechanisms and possible therapies, and the significance of genetic modifiers of the disease identified in these models in delineating the underlying molecular mechanisms.

## Introduction

Gain-of-function (GOF) mutations in the human rhomboid 5 homolog 2 (*RHBDF2*) gene constitutively activate epidermal growth factor receptor (EGFR) signaling to cause tylosis with esophageal cancer syndrome (TOC; OMIM: 148500) (Blaydon et al., 2012; Saarinen et al., 2012; Mokoena et al., 2018). *RHBDF2* encodes the highly conserved, seven-transmembrane, rhomboid protein family member RHBDF2 (Freeman, 2014). Although the mechanisms underlying RHBDF2-mediated EGFR signaling are still emerging, gaining insights into these mechanisms will contribute substantially to the discovery of unique drug targets for treating TOC and potentially other related skin diseases.

Substantial literature suggests that RHBDF2 regulates the EGFR signaling pathway and its downstream signaling events, including rapid wound healing and epithelial tumorigenesis, through enhanced secretion of the EGFR ligand amphiregulin (AREG) (Brooke et al., 2014; Hosur et al., 2014, 2017a). AREG is synthesized as a pro-protein that requires conversion to an active protein by a disintegrin and metalloprotease 17 (ADAM17), in a process known as ectodomain shedding (Blobel, 2005). Two models offer possible explanations of the role of RHBDF2 in regulating AREG secretion. The first proposes that RHBDF2 directly interacts with ADAM17 and regulates its maturation, trafficking, and ectodomain shedding activity. According to the second model, RHBDF2 regulates AREG secretion through ADAM17, as in the first model, but importantly, it does so without altering ADAM17 sheddase activity. Further studies are needed to establish specific biological functions and properties of RHBDF2 in mediating AREG secretion in health and disease.

Currently there is no cure for TOC. To find a cure, two hurdles must be addressed immediately. First, mouse models of human TOC are critically needed to identify the relevant cell types, and to understand the pathogenesis and how to approach therapeutic treatment. Second, it is imperative to better understand the mechanisms underlying RHBDF2-mediated EGFR signaling by corroborating the significance of *in vitro* mechanistic findings in mouse models of human TOC.

## Murine Models

**FIGURE 1:**
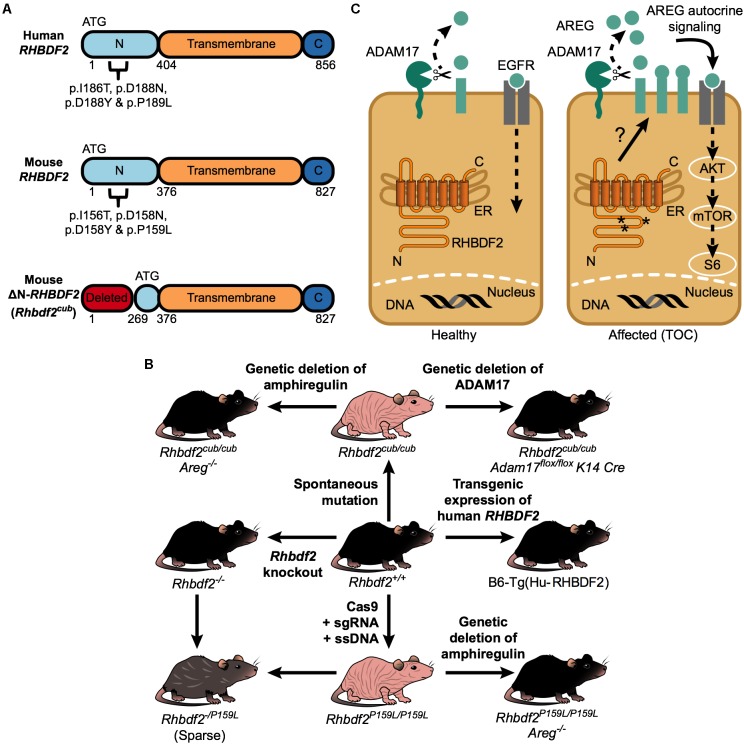
RHBDF2-regulated epidermal growth factor receptor signaling in TOC. **(A)** A schematic of the human RHBDF2 protein showing all four missense mutations in the cytosolic N-terminus domain that underlie human TOC (top); a schematic of the mouse RHBDF2 protein showing the analogous human TOC missense mutations (middle); a schematic of the truncated mouse RHBDF2 protein, encoded by the *Rhbdf2^cub^* gene, showing deletion of the cytosolic N-terminus domain (bottom). Notably, in the truncated protein, the cytosolic N-terminus domain still retains amino acids 269–375. **(B)** Diagram showing that both spontaneous (*Rhbdf2^cub/cub^*) and genetically engineered (*Rhbdf2*^*P*159*L*/*P*159*L*^) mouse models of human TOC exhibit a loss-of-hair phenotype. First, to validate that *Rhbdf2^cub/cub^* and *Rhbdf2*^*P*159*L*/*P*159*L*^ are gain-of-function (GOF) mutations rather than null mutations, we generated *Rhbdf2* knockout *(Rhbdf2*^−/−^*)* mice and observed that *Rhbdf2*^−/−^ mice present with a normal hair coat, similarly to the wild-type (*Rhbdf2*^+/+^) mice, but very different from *Rhbdf2^cub/cub^* or *Rhbdf2*^*P*159*L*/*P*159*L*^ mice. Second, to validate that *Rhbdf2^cub^* and *Rhbdf2*^*P*159*L*^ are mutant alleles of the *Rhbdf2* gene, we generated *Rhbdf2^cub/−^* and *Rhbdf2*^*P*159*L*/−^ mice, which exhibit a sparse hair coat phenotype, suggesting that *Rhbdf2^cub^* and *Rhbdf2*^*P*159*L*^ are mutant alleles of the *Rhbdf2* gene. Third, we identified AREG and ADAM17 as genetic modifiers of the TOC phenotype; genetic deletion of *Areg* or *Adam17* in both *Rhbdf2^cub/cub^* and *Rhbdf2*^*P*159*L*/*P*159*L*^ mice restores the normal skin phenotype in these mice, suggesting that AREG and ADAM17 are essential mediators of the TOC phenotype. Lastly, we generated a B6-Tg(Hu-RHBDF2) mouse strain using a bacterial artificial chromosome (BAC) containing the human RHBDF2 gene, which, similarly to *Rhbdf2*^+/+^ and *Rhbdf2*^−/−^ mice, exhibits a normal hair coat. Together, our results suggest that neither loss of RHBDF2 nor overexpression of RHBDF2, but GOF mutations, such as *Rhbdf2^cub^* and *Rhbdf2*^*P*159*L*^, induce an overt “EGFR hyperactive” phenotype to cause TOC. **(C)** RHBDF2 GOF mutations enhance AREG secretion through ADAM17 in a tissue-specific manner, leading to EGFR hyperactivation and in turn TOC.

Mouse models of human disease have long served a valuable role in biomedical research. In recent years, we have generated and validated mice carrying the human TOC disease mutation p.P189L (Hosur et al., 2017a; **Figure 1A**, top panel, **Figure 1B**), characterized a spontaneous mutation that results in a TOC phenotype (Hosur et al., 2014), and used these mouse models of TOC in studies in which we identified AREG as a functional driver of the disease (Hosur et al., 2014, 2017a); established an essential role for ADAM17 in mediating the disease (Hosur et al., 2018); and, most recently, demonstrated that RHBDF2-AREG-EGFR signaling, and not the immune system or surrounding microenvironment, plays a role in mediating the disease (Hosur et al., 2017b). These and other murine models of TOC (**Table 1**) will be useful for methodically studying the molecular pathways underlying TOC in further detail, for investigating the molecular pathways underlying related proliferative skin diseases, and for testing novel and existing therapeutics.

**Table 1:** Mouse models of the human skin disease tylosis with esophageal cancer syndrome.

*Strain*	Background	Description	Reference
*Rhbdf2^cub/cub^*	C57BL/6J	• Augmented secretion of amphiregulin (AREG).	Johnson et al., 2003; Hosur et al., 2014
		• Aberrant EGFR signaling.	
		• Hyperplasia, hyperkeratosis, alopecia, rapid cutaneous wound healing, and increased susceptibility to epithelial cancers.	
*Rhbdf2^P159/P159L^*	C57BL/6J	• Augmented secretion of AREG.	Hosur et al., 2017a
		• Aberrant EGFR signaling.	
		• Hyperplasia, hyperkeratosis, alopecia, and rapid cutaneous wound healing.	
*Rhbdf2^uncv/uncv^*	BALB/c	• The uncovered (*Rhbdf2^uncv/uncv^*) spontaneous mouse mutation, similarly to the *Rhbdf2^cub/cub^* mutation, results in the loss of the cytosolic N-terminal domain of RHBDF2, and consequently a loss-of-hair phenotype.	Li et al., 1999; Leilei et al., 2014
*Rhbdf2*^−/−^	C57BL/6 (?)	• Forepaws and hind paws lack the normal epidermal thickening and hyperpigmentation of the footpads.	Maruthappu et al., 2017
		• Reduced keratin 16 expression in the footpads.	
		• Reduced stimulated secretion of EGFR ligands, including AREG, heparin-binding EGF (HB-EGF), and transforming growth factor alpha (TGFA).	
*Rhbdf2^cub/cub^ Areg^Mcub/Mcub^*	C57BL/6J	• A point mutation in the *Areg* gene, resulting in a premature stop codon.	Johnson et al., 2003; Hosur et al., 2014; Siggs et al., 2014
		• Amphiregulin-null mice. No detectable *Areg* mRNA or protein.	
		• Wavy hair coat owing to certain defects in EGFR signaling.	
*Rhbdf2^P159/P159L^ Areg^Mcub/Mcub^*	C57BL/6J	• A point mutation in the *Areg* gene, resulting in a premature stop codon.	Hosur et al., 2017a
		• Amphiregulin-null mice. No detectable *Areg* mRNA or protein.	
		• Normal hair coat. No apparent defects in EGFR signaling.	
*Rhbdf2^cub/cub^ Apc^Min/+^*	C57BL/6J	• Increases adenoma formation and reduces survival in *Apc^Min/+^* mice.	Hosur et al., 2014
MRL/MpJ-*Rhbdf2^cub/cub^*	MRL/MpJ	• Augmented secretion of AREG.	Hosur et al., 2017b
		• Aberrant EGFR signaling.	
		• Hyperplasia, hyperkeratosis, alopecia, and rapid cutaneous wound healing.	
*Rhbdf2^cub/cub^ Adam17^flox/flox^ K14-Cre*	Mixed genetic background	• *Rhbdf2^cub/cub^* mice lacking ADAM17 specifically in the skin.	Hosur et al., 2018
		• Loss of AREG secretion.	
		• Full hair coat, and loss of rapid wound-healing phenotype.	
		• Dermatitis and myeloproliferative disease in *Rhbdf2^cub/cub^* mice lacking ADAM17 specifically in the skin.	

### A Spontaneous Mouse Mutation: Curly Bare (*Rhbdf2^cub/cub^*)

Spontaneous mouse mutations have been a valuable source of animal models for studying heritable human diseases, both for discovering the role of gene function and for understanding the biological pathways involved. Johnson et al. (2003) described a spontaneous recessive mouse mutation, named curly bare (*cub*) that maps to the distal region of Chr 11 and exhibits a hairless phenotype (**Figure 1B**). Hosur et al. (2014) reported that the *cub* mouse mutation is an unusually large (12,681 bp) deletion in the *Rhbdf2* gene, resulting in the loss of exons 2–6. We also showed that even though the normal translation initiation site (ATG) in exon 3 is missing, there is translation from a downstream initiation site in exon 8, leading to a ∼63.5-kDa truncated protein rather than a full-length ∼100-kDa protein (**Figure 1A**; bottom panel). Histological analyses of skin sections from *Rhbdf2^cub/cub^* mice revealed epidermal hyperplasia, enlarged sebaceous glands, alopecia, and rapid cutaneous wound healing (data not shown). Additionally, embryonic fibroblasts isolated from *Rhbdf2^cub/cub^* mice exhibited a hyperactive EGFR signaling phenotype mediated by enhanced secretion of AREG (data not shown). Surprisingly, *Rhbdf2* knockout (*Rhbdf2*^−/−^) mice appeared normal (**Figure 1B**), with no skin or rapid cutaneous wound-healing phenotype (Hosur et al., 2014; Siggs et al., 2014), suggesting that *Rhbdf2^cub/cub^* is a GOF mutation rather than a null mutation.

Next, to test the influence of mouse strain background on the *Rhbdf2^cub/cub^* phenotype, we backcrossed the *Rhbdf2^cub/cub^* mutation from the C57BL/6J background onto the MRL/MpJ background for more than 20 generations, creating a true congenic strain, MRL/MpJ-*Rhbdf2^cub/cub^* mice (Hosur et al., 2017b). We observed not only the rapid cutaneous wound-healing phenotype but also the hyperproliferative-skin phenotype in these mice, suggesting that the *Rhbdf2^cub/cub^* mutation results in a similar phenotype regardless of the mouse inbred strain background.

Tylosis is strongly associated with esophageal squamous cell carcinoma (Howel-Evans et al., 1958; Harper et al., 1970; Ellis et al., 1994; Hennies et al., 1995); however, there is no spontaneous incidence of esophageal cancer in *Rhbdf2^cub/cub^* mice. Because cigarette smoking and alcohol consumption are known risk factors for esophageal squamous cell carcinoma (Montgomery et al., 2014), we reasoned that the *Rhbdf2^cub^* mutation could increase the susceptibility to epithelial cancers and tested the role of RHBDF2 in adenoma formation in *Apc^Min/+^* mice, a mouse model of human colon cancer. We found that a single allele of *Rhbdf2^cub^* can accelerate polyp formation and reduce the survival rates of *Apc^Min/+^* mice (data not shown) (Hosur et al., 2014). Histological analysis revealed significantly increased tumor size and an increased number of polyps in *Apc^Min/+^ Rhbdf2^+/cub^* mice compared with *Apc^Min/+^ Rhbdf2^+/+^* mice. Moreover, stimulated secretion of AREG was significantly increased in intestinal epithelial cells isolated from *Apc^Min/+^ Rhbdf2^+/cub^* mice compared with *Apc^Min/+^ Rhbdf2^+/+^* mice. Together, these data suggest that GOF mutations in RHBDF2 accelerate tumor growth with an associated increase in AREG secretion, suggesting a critical role for RHBDF2 in controlling tumor growth through enhanced secretion of AREG.

### A Genetically Engineered Mouse Model: *Rhbdf2^P159L/P159L^*

The capacity of CRISPR/Cas9 technology to be used to recapitulate patient variants in mouse models for various human diseases is well established (Birling et al., 2017). Using CRISPR/Cas9-mediated targeting and homology-directed repair in C57BL/6J zygotes, we recently generated mice carrying the human TOC mutation p.P189L (p.P159L in mice) in *RHBDF2* (**Figure 1A**, top and middle panels, **Figure 1B**) and showed that the *Rhbdf2*^*P*159*L*^ GOF mutation enhances AREG secretion to cause alopecia, rapid cutaneous wound healing, hyperplasia, and hyperkeratosis (Hosur et al., 2017a). Also, immunohistochemical analyses of skin sections from *Rhbdf2^+/+^* and *Rhbdf2^P159L/P159L^* mice revealed a considerable increase in the activity of downstream effectors of the epidermal EGFR signaling pathway, including phospho-ERK1/2 and phospho-mTOR, in *Rhbdf2^P159L/P159L^* mice. Together, these data suggest that, analogous to the phenomena in human TOC mutations, *Rhbdf2^P159L/P159L^* is a GOF mutation in the mouse *Rhbdf2* gene, and that these GOF mutations enhance secretion of AREG and lead to constitutive activation of EGFR signaling to cause TOC.

### *Rhbdf2* Loss-of-Function Mice: *Rhbdf2*^−/−^

Since GOF mutations in RHBDF2 cause skin hyperkeratosis and hyperplasia (Blaydon et al., 2012; Saarinen et al., 2012; Mokoena et al., 2018), Maruthappu et al. (2017) examined whether loss of RHBDF2 has any effect on skin thickness. The authors examined hematoxylin and eosin-stained skin sections of adult *Rhbdf2*^−/−^ and *Rhbdf2^+/+^* mice and reported that both the forepaws and hind paws, but not the back skin, of *Rhbdf2*^−/−^ mice present with an epidermis thinner than that of *Rhbdf2^+/+^* mice. Additionally, the authors showed that the thinner epidermis in *Rhbdf2*^−/−^ mice is associated with reduced expression of cytoskeletal stress-associated protein keratin 16 (K16). Because of the reduced K16 expression associated with loss of RHBDF2 and a thinner epidermis, the authors examined K16 expression in the skin of human TOC patients using immunohistochemistry and observed a considerable increase in K16 expression. Collectively, these findings suggest that loss of RHBDF2 results in a thinner epidermis, and that, correspondingly, GOF mutations in RHBDF2, such as those observed in TOC, result in epidermal thickening through interaction with and regulation of K16.

## Potential Mechanisms

### Amphiregulin as a Functional Driver of the Disease

Genetic modifier genes can accelerate disease progression, slow down disease progression, or completely reverse a disease, and thus provide valuable information regarding the underlying biological pathways and the potential pharmaceutical targets. The *Rhbdf2^cub^* mouse modifier gene Modifier of cub phenotype (*Mcub*) was mapped to a position that coincides with a cluster of four EGFR ligand-encoding genes on chromosome 5 (Johnson et al., 2003). A single *Mcub* allele in combination with the *Rhbdf2^cub/cub^* genotype restores a full, wavy hair coat in *Rhbdf2^cub/cub^* mice. *Mcub* was later identified as a point mutation in the *Areg* gene that results in a premature stop codon, which in turn results in the absence of any detectable *Areg* mRNA or protein (Hosur et al., 2014). The rapid cutaneous wound-healing and alopecia phenotype observed in *Rhbdf2^cub/cub^* mice is lost when a single copy of the *Mcub* allele is present in combination with the *Rhbdf2^cub/cub^* genotype. Concordantly, in *Rhbdf2^P159L/P159L^* mice, genetic deletion of *Areg* restores the normal skin phenotype (Hosur et al., 2017a), suggesting that AREG mediates the hyperplasia, hyperkeratosis, alopecia, and rapid wound-healing phenotypes in *Rhbdf2^P159L/P159L^* and *Rhbdf2^cub/cub^* mice.

### The Metalloprotease ADAM17 Is Essential for Shedding of Amphiregulin

The metalloprotease ADAM17 is the major ectodomain sheddase of AREG (Sunnarborg et al., 2002; Sahin et al., 2004; Sternlicht et al., 2005). However, in studies using *Rhbdf2^cub/cub^* mice, we (Hosur et al., 2014) and others (Leilei et al., 2014; Siggs et al., 2014) found that ADAM17 activity is reduced in *Rhbdf2^cub/cub^* mice, suggesting that RHBDF2 regulates secretion of AREG independently of ADAM17 activity. Thus, to examine whether RHBDF2 participates directly in shedding of EGFR ligands, similarly to ADAM17, we generated *Rhbdf2^cub/cub^* mice with ADAM17-deficient keratinocytes (*Rhbdf2^cub/cub^ Adam17^flox/flox^ K14-Cre*) and studied their phenotype (Hosur et al., 2018). We found that ADAM17 deficiency in the skin of *Rhbdf2^cub/cub^* mice restored a full hair coat and impaired the rapid wound-healing phenotype observed in *Rhbdf2^cub/cub^* mice, demonstrating that ADAM17 is the major ectodomain sheddase of AREG and that RHBDF2 does not directly participate in the shedding of AREG. In addition, *Rhbdf2^cub/cub^* mouse embryonic keratinocytes lacking ADAM17 failed to secrete AREG both under stimulated and unstimulated conditions, further demonstrating that ADAM17 is the principal sheddase of AREG and, importantly, that RHBDF2 does not directly participate in shedding of AREG (**Figure 1C**).

### Tissue-Specific Role of the RHBDF2-AREG-ADAM17-EGFR Pathway

The immune system plays a key role in the pathogenesis of many proliferative skin diseases, including psoriasis and atopic dermatitis (Reich, 2012; Czarnowicki et al., 2014). To determine whether the hyperproliferative-skin and rapid-wound-healing phenotypes mediated by RHBDF2-AREG in TOC are tissue-specific and persist independently of the immune system, we performed bone marrow and reciprocal skin graft experiments (Hosur et al., 2017b). Bone marrow transfer from C57BL/6J(B6)-*Rhbdf2^cub/cub^* donor mice to B6 recipient mice failed to transfer the proliferative-skin and cutaneous regenerative phenotype in B6 mice, suggesting that the surrounding microenvironment or the immune system are unlikely to contribute to the TOC phenotype. Moreover, reciprocal skin grafts—from B6 mice to the dorsal skin of B6-*Rhbdf2^cub/cub^* mice and from B6-*Rhbdf2^cub/cub^* mice to the dorsal skin of B6 mice—maintained the phenotype of the donor mice. Together, these findings suggest a tissue-specific function of the RHBDF2-AREG-ADAM17-EGFR pathway in TOC.

### Revisiting the Role of RHBDF2 in Regulating AREG Secretion

The mechanism by which RHBDF2 regulates AREG secretion is unclear. Substantial literature suggests that RHBDF2 regulates ectodomain shedding of AREG secretion by controlling the sheddase activity of ADAM17, the principle sheddase of AREG. This model is based primarily on the hypothesis that RHBDF2 is essential for not only the maturation and trafficking but also the activation of ADAM17 (Adrain et al., 2012; McIlwain et al., 2012; Christova et al., 2013; Li et al., 2015; Cavadas et al., 2017; Grieve et al., 2017). In further support of this model, it has been shown that lack of RHBDF2 significantly decreases ADAM17-dependent AREG secretion after stimulation with the protein kinase C stimulant PMA, whereas GOF *RHBDF2* mutations such as those observed in human TOC enhance ADAM17 sheddase activity to significantly increase AREG secretion (Brooke et al., 2014). Consistent with this model, Maney et al. (2015) recently showed *in vitro* that GOF mutations in RHBDF2 enhance ADAM17 sheddase activity. Furthermore, it has been suggested that RHBDF1, a paralog of RHBDF2, can compensate for the loss of RHBDF2 in regulating ADAM17 maturation, trafficking, and activity (Issuree et al., 2013).

Conversely, the following evidence argues against a role for RHBDF1/RHBDF2 in regulating ADAM17 trafficking and maturation or activity to drive AREG secretion. Strong *in vivo* evidence includes the finding that mice overexpressing ADAM17 are viable and exhibit no overt phenotype (Yoda et al., 2013). Concordantly, overexpression of ADAM17 *in vivo* does not elicit any enhanced ADAM17 sheddase activity, or a significant increase in secretion of AREG or other ADAM17 substrates (Fukaya et al., 2013; Yoda et al., 2013). In support of these observations, Dang et al. (2013) demonstrated *in vitro* that phorbol-ester-stimulated shedding of EGF family members and ADAM17 substrates AREG, HB-EGF, and TGFα occurs independently of major changes in ADAM17 sheddase activity. Studies using *Rhbdf1* knockout and/or *Rhbdf1*/*Rhbdf2* double knockout mice suggest that it is unlikely that RHBDF1 is involved in regulating ADAM17 activity or compensating for its loss. Neither *Rhbdf1* knockout mice nor *Rhbdf1*/*Rhbdf*2 double knockout mice phenocopy *Adam17* knockout mice; whereas as *Rhbdf1* knockout and *Rhbdf1*/*Rhbdf2* double knockout mice exhibit postnatal and embryonic lethality (Christova et al., 2013), respectively, *Adam17* knockout mice exhibit perinatal lethality with an “open eyelids at birth” phenotype and hair-follicle defects (Peschon et al., 1998). Together, these *in vitro* and *in vivo* findings strongly argue against a role for RHBDF1 and RHBDF2 in either maturation or trafficking of ADAM17, or regulation of its activity, to drive AREG secretion. We propose that RHBDF2 likely regulates the availability of AREG without directly influencing ADAM17 maturation or trafficking, or sheddase activity (**Figure 1C**, right panel).

## Conclusion

As mice and humans share between 95 and 98% of their genes, mouse models of human skin diseases have been valuable resources for studying the normal biological processes involved in skin function, understanding the molecular mechanism causing the diseases, identifying potential targets for intervention, and testing therapeutics. While current murine models of human TOC, both spontaneous mutant and genetically engineered mice, do not recapitulate all characteristics of the human disease, these models have yielded valuable insights into disease mechanisms, including the identification of AREG as a functional driver of the disease, verification of the essential role of metalloprotease ADAM17 in shedding of AREG, demonstration of the susceptibility of mice carrying *Rhbdf2* GOF mutations to epithelial cancer, and determination of the tissue-specific role of the RHBDF2-AREG-ADAM17-EGFR pathway. In addition, by further leveraging these murine models in future studies, researchers can continue to generate and test mechanistic hypotheses, and study the physiological and pathogenic molecular machinery underlying the role of the RHBDF2-regulated EGFR signaling pathway in TOC, and in other skin diseases and epithelial cancers.

## Author Contributions

VH designed the experiments, involved in generation and characterization of mouse models, wrote and reviewed the manuscript. MF, BL, and LB involved in generation and characterization of mouse models. LS and MW designed, wrote, and reviewed the manuscript. All authors read and approved the final manuscript.

## Conflict of Interest Statement

The authors declare that the research was conducted in the absence of any commercial or financial relationships that could be construed as a potential conflict of interest.

## References

[B1] Adrain C. Zettl M. Christova Y. Taylor N. Freeman M. (2012). Tumor necrosis factor signaling requires iRhom2 to promote trafficking and activation of TACE. *Science* 335 225–228. 10.1126/science.1214400 22246777 PMC3272371

[B2] Birling M. C. Herault Y. Pavlovic G. (2017). Modeling human disease in rodents by CRISPR/Cas9 genome editing. *Mamm. Genome* 28 291–301. 28677007 10.1007/s00335-017-9703-x PMC5569124

[B3] Blaydon D. C. Etheridge S. L. Risk J. M. Hennies H. C. Gay L. J. Carroll R. (2012). RHBDF2 mutations are associated with tylosis, a familial esophageal cancer syndrome. *Am. J. Hum. Genet.* 90 340–346. 10.1016/j.ajhg.2011.12.008 22265016 PMC3276661

[B4] Blobel C. P. (2005). ADAMs: key components in EGFR signalling and development. *Nat. Rev. Mol. Cell Biol.* 6 32–43. 10.1038/nrm1548 15688065

[B5] Brooke M. A. Etheridge S. L. Kaplan N. Simpson C. O’Toole E. A. Ishida-Yamamoto A. (2014). iRHOM2-dependent regulation of ADAM17 in cutaneous disease and epidermal barrier function. *Hum. Mol. Genet.* 23 4064–4076. 10.1093/hmg/ddu120 24643277 PMC4110483

[B6] Cavadas M. Oikonomidi I. Gaspar C. J. Burbridge E. Badenes M. Felix I. (2017). Phosphorylation of iRhom2 controls stimulated proteolytic shedding by the metalloprotease ADAM17/TACE. *Cell Rep.* 21 745–757. 10.1016/j.celrep.2017.09.074 29045841 PMC5656746

[B7] Christova Y. Adrain C. Bambrough P. Ibrahim A. Freeman M. (2013). Mammalian iRhoms have distinct physiological functions including an essential role in TACE regulation. *EMBO Rep.* 14 884–890. 10.1038/embor.2013.128 23969955 PMC3807218

[B8] Czarnowicki T. Krueger J. G. Guttman-Yassky E. (2014). Skin barrier and immune dysregulation in atopic dermatitis: an evolving story with important clinical implications. *J. Allergy Clin. Immunol. Pract.* 2 371–379. 25017523 10.1016/j.jaip.2014.03.006

[B9] Dang M. Armbruster N. Miller M. A. Cermeno E. Hartmann M. Bell G. W. (2013). Regulated ADAM17-dependent EGF family ligand release by substrate-selecting signaling pathways. *Proc. Natl. Acad. Sci. U.S.A.* 110 9776–9781. 10.1073/pnas.1307478110 23720309 PMC3683718

[B10] Ellis A. Field J. K. Field E. A. Friedmann P. S. Fryer A. Howard P. (1994). Tylosis associated with carcinoma of the oesophagus and oral leukoplakia in a large Liverpool family–a review of six generations. *Eur. J. Cancer B Oral Oncol.* 30b 102–112. 10.1016/0964-1955(94)90061-2 8032299

[B11] Freeman M. (2014). The rhomboid-like superfamily: molecular mechanisms and biological roles. *Annu. Rev. Cell Dev. Biol.* 30 235–254. 10.1146/annurev-cellbio-100913-012944 25062361

[B12] Fukaya S. Matsui Y. Tomaru U. Kawakami A. Sogo S. Bohgaki T. (2013). Overexpression of TNF-alpha-converting enzyme in fibroblasts augments dermal fibrosis after inflammation. *Lab. Invest.* 93 72–80. 10.1038/labinvest.2012.153 23147225

[B13] Grieve A. G. Xu H. Kunzel U. Bambrough P. Sieber B. Freeman M. (2017). Phosphorylation of iRhom2 at the plasma membrane controls mammalian TACE-dependent inflammatory and growth factor signalling. *eLife* 6:e23968. 28432785 10.7554/eLife.23968 PMC5436907

[B14] Harper P. S. Harper R. M. Howel-Evans A. W. (1970). Carcinoma of the oesophagus with tylosis. *Q. J. Med.* 39 317–333. 5478505

[B15] Hennies H. C. Hagedorn M. Reis A. (1995). Palmoplantar keratoderma in association with carcinoma of the esophagus maps to chromosome 17q distal to the keratin gene cluster. *Genomics* 29 537–540. 8666405 10.1006/geno.1995.9971

[B16] Hosur V. Farley M. L. Burzenski L. M. Shultz L. D. Wiles M. V. (2018). ADAM17 is essential for ectodomain shedding of the EGF-receptor ligand amphiregulin. *FEBS Open Bio* 8 702–710. 10.1002/2211-5463.12407 PMC5881543 29632822

[B17] Hosur V. Johnson K. R. Burzenski L. M. Stearns T. M. Maser R. S. Shultz L. D. (2014). Rhbdf2 mutations increase its protein stability and drive EGFR hyperactivation through enhanced secretion of amphiregulin. *Proc. Natl. Acad. Sci. U.S.A.* 111 E2200–E2209. 24825892 10.1073/pnas.1323908111 PMC4040562

[B18] Hosur V. Low B. E. Shultz L. D. Wiles M. V. (2017a). Genetic deletion of amphiregulin restores the normal skin phenotype in a mouse model of the human skin disease tylosis. *Biol. Open* 6 1174–1179. 28655741 10.1242/bio.026260 PMC5576083

[B19] Hosur V. Lyons B. L. Burzenski L. M. Shultz L. D. (2017b). Tissue-specific role of RHBDF2 in cutaneous wound healing and hyperproliferative skin disease. *BMC Res. Notes* 10:573. 10.1186/s13104-017-2899-8 29116018 PMC5678570

[B20] Howel-Evans W. Mc C. R. Clarke C. A. Sheppard P. M. (1958). Carcinoma of the oesophagus with keratosis palmaris et plantaris (tylosis): a study of two families. *Q. J. Med.* 27 413–429. 13579162

[B21] Issuree P. D. Maretzky T. McIlwain D. R. Monette S. Qing X. Lang P. A. (2013). iRHOM2 is a critical pathogenic mediator of inflammatory arthritis. *J. Clin. Invest.* 123 928–932. 10.1172/JCI66168 23348744 PMC3561822

[B22] Johnson K. R. Lane P. W. Cook S. A. Harris B. S. Ward-Bailey P. F. Bronson R. T. (2003). Curly bare (cub), a new mouse mutation on chromosome 11 causing skin and hair abnormalities, and a modifier gene (mcub) on chromosome 5. *Genomics* 81 6–14. 10.1016/S0888-7543(02)00013-7 12573256

[B23] Leilei Y. Bing L. Yang L. Shaoxia W. Yuan X. Dongping W. (2014). iRhom2 mutation leads to aberrant hair follicle differentiation in mice. *PLoS One* 9:e115114. 10.1371/journal.pone.0115114 25546423 PMC4278852

[B24] Li S. R. Wang D. P. Yu X. L. Ge B. S. Wang C. E. Lu Y. F. (1999). Uncv (uncovered): a new mutation causing hairloss on mouse chromosome 11. *Genet. Res.* 73 233–238. 10.1017/S0016672399003808 10425919

[B25] Li X. Maretzky T. Weskamp G. Monette S. Qing X. Issuree P. D. (2015). iRhoms 1 and 2 are essential upstream regulators of ADAM17-dependent EGFR signaling. *Proc. Natl. Acad. Sci. U.S.A.* 112 6080–6085. 10.1073/pnas.1505649112 25918388 PMC4434755

[B26] Maney S. K. McIlwain D. R. Polz R. Pandyra A. A. Sundaram B. Wolff D. (2015). Deletions in the cytoplasmic domain of iRhom1 and iRhom2 promote shedding of the TNF receptor by the protease ADAM17. *Sci. Signal.* 8:ra109. 10.1126/scisignal.aac5356 26535007 PMC7202466

[B27] Maruthappu T. Chikh A. Fell B. Delaney P. J. Brooke M. A. Levet C. (2017). Rhomboid family member 2 regulates cytoskeletal stress-associated Keratin 16. *Nat. Commun.* 8:14174. 10.1038/ncomms14174 28128203 PMC5290154

[B28] McIlwain D. R. Lang P. A. Maretzky T. Hamada K. Ohishi K. Maney S. K. (2012). iRhom2 regulation of TACE controls TNF-mediated protection against Listeria and responses to LPS. *Science* 335 229–232. 10.1126/science.1214448 22246778 PMC4250273

[B29] Mokoena T. Smit J. G. M. Karusseit V. O. Dorfling C. M. van Rensburg E. J. (2018). Tylosis associated with squamous cell carcinoma of the oesophagus (TOC): report of an African family with a novel RHBDF2 variant. *Clin. Genet.* 93 1114–1116. 29372562 10.1111/cge.13161

[B30] Montgomery E. Basman F. Brennan P. Malekzadeh R. (2014). “Oesophageal cancer,” in *World Cancer Report* eds Stewart B. W. Wild C. P., (Geneva: World Health Organization), 374–382.

[B31] Peschon J. J. Slack J. L. Reddy P. Stocking K. L. Sunnarborg W. S. Lee D. C. (1998). An essential role for ectodomain shedding in mammalian development. *Science* 282 1281–1284. 10.1126/science.282.5392.1281 9812885

[B32] Reich K. (2012). The concept of psoriasis as a systemic inflammation: implications for disease management. *J. Eur. Acad. Dermatol. Venereol.* 26(Suppl. 2), 3–11. 10.1111/j.1468-3083.2011.04410.x 22356630

[B33] Saarinen S. Vahteristo P. Lehtonen R. Aittomaki K. Launonen V. Kiviluoto T. (2012). Analysis of a Finnish family confirms RHBDF2 mutations as the underlying factor in tylosis with esophageal cancer. *Fam. Cancer* 11 525–528. 10.1007/s10689-012-9532-8 22638770

[B34] Sahin U. Weskamp G. Kelly K. Zhou H. M. Higashiyama S. Peschon J. (2004). Distinct roles for ADAM10 and ADAM17 in ectodomain shedding of six EGFR ligands. *J. Cell Biol.* 164 769–779. 10.1083/jcb.200307137 14993236 PMC2172154

[B35] Siggs O. M. Grieve A. Xu H. Bambrough P. Christova Y. Freeman M. (2014). Genetic interaction implicates iRhom2 in the regulation of EGF receptor signalling in mice. *Biol. Open* 3 1151–1157. 25395669 10.1242/bio.201410116 PMC4265752

[B36] Sternlicht M. D. Sunnarborg S. W. Kouros-Mehr H. Yu Y. Lee D. C. Werb Z. (2005). Mammary ductal morphogenesis requires paracrine activation of stromal EGFR via ADAM17-dependent shedding of epithelial amphiregulin. *Development* 132 3923–3933. 10.1242/dev.01966 16079154 PMC2771180

[B37] Sunnarborg S. W. Hinkle C. L. Stevenson M. Russell W. E. Raska C. S. Peschon J. J. (2002). Tumor necrosis factor-alpha converting enzyme (TACE) regulates epidermal growth factor receptor ligand availability. *J. Biol. Chem.* 277 12838–12845. 10.1074/jbc.M112050200 11823465

[B38] Yoda M. Kimura T. Tohmonda T. Morioka H. Matsumoto M. Okada Y. (2013). Systemic overexpression of TNFalpha-converting enzyme does not lead to enhanced shedding activity in vivo. *PLoS One* 8:e54412. 10.1371/journal.pone.0054412 23342154 PMC3544834

